# Phenotypic variation and genotype-phenotype discordance in canine cone-rod dystrophy with an *RPGRIP1* mutation

**Published:** 2009-11-11

**Authors:** Keiko Miyadera, Kumiko Kato, Jesús Aguirre-Hernández, Tsuyoshi Tokuriki, Kyohei Morimoto, Claudia Busse, Keith Barnett, Nigel Holmes, Hiroyuki Ogawa, Nobuo Sasaki, Cathryn S. Mellersh, David R. Sargan

**Affiliations:** 1 Department of Veterinary Medicine, University of Cambridge, Madingley Road, Cambridge, United Kingdom; 2 Department of Veterinary Medical Science, Graduate School of Agricultural and Life Sciences, University of Tokyo, Tokyo, Japan; 3 Comparative Ophthalmology Unit, Animal Health Trust, Lanwades Park, Kentford, Newmarket, Suffolk, United Kingdom; 4 Centre for Preventive Medicine, Animal Health Trust, Lanwades Park, Kentford, Newmarket, Suffolk, United Kingdom; 5 Japan Animal Medical Referral Center, Kanagawa, Japan

## Abstract

**Purpose:**

Previously, a 44 bp insertion in exon 2 of retinitis pigmentosa GTPase interacting protein 1 (*RPGRIP1)* was identified as the cause of cone-rod dystrophy 1 (*cord1*), a recessive form of progressive retinal atrophy (PRA) in the Miniature Longhaired Dachshund (MLHD), a dog model for Leber congenital amaurosis. The *cord1* locus was mapped using MLHDs from an inbred colony with a homogeneous early onset disease phenotype. In this paper, the MLHD pet population was studied to investigate phenotypic variation and genotype-phenotype correlation. Further, the *cord1* locus was fine-mapped using PRA cases from the MLHD pet population to narrow the critical region. Other dog breeds were also screened for the *RGPRIP1* insertion.

**Methods:**

This study examined phenotypic variation in an MLHD pet population that included 59 sporadic PRA cases and 18 members of an extended family with shared environment and having six PRA cases. Ophthalmologic evaluations included behavioral abnormalities, responses to menace and light, fundoscopy, and electroretinography (ERG). The *RPGRIP1* insertion was screened for in all cases and 200 apparently normal control MLHDs and in 510 dogs from 66 other breed. To fine-map the *cord1* locus in the MLHD, 74 PRA cases and 86 controls aged 4 years or more were genotyped for 24 polymorphic markers within the previously mapped *cord1* critical region of 14.15 Mb.

**Results:**

Among sporadic PRA cases from the MLHD pet population, the age of onset varied from 4 months to 15 years old; MLHDs from the extended family also showed variable onset and rate of progression. Screening for the insertion in *RPGRIP1* identified substantial genotype-phenotype discordance: 16% of controls were homozygous for the insertion (*RPGRIP1*^−/−^), while 20% of PRA cases were not homozygous for it. Four other breeds were identified to carry the insertion including English Springer Spaniels and Beagles with insertion homozygotes. The former breed included both controls and PRA cases, yet in the latter breed, cone ERG was undetectable in two dogs with no clinically apparent visual dysfunction. Notably, the insertion in the Beagles was a longer variant of that seen in the other breeds. Fine-mapping of the *cord1* locus narrowed the critical region on CFA15 from 14.15 Mb to 1.74 Mb which still contains the *RPGRIP1* gene.

**Conclusions:**

Extensive phenotypic variations of onset age and progression rate were observed in PRA cases of the MLHD pet population. The insertion in *RPGRIP1* showed the strongest association with the disease, yet additional as well as alternative factors may account for the substantial genotype-phenotype discordance.

## Introduction

Hereditary blindness caused by retinal degeneration is among the best-characterized genetic conditions in the dog, both clinically and genetically. Progressive retinal atrophy (PRA) is a group of inherited diseases of the retina, causing gradual vision loss leading to blindness in a variety of dog breeds. Typically, each affected breed expresses a breed-specific clinical phenotype with a characteristic mode of inheritance, age of onset, rate of progression, and pathogenesis [1]. The various phenotypes are related to distinct breed-specific mutations in different genes involved in visual function. To date, 15 mutations in 11 genes causing PRA in 34 dog breeds and breed subtypes have been identified.

The dog retina has many similarities to the human retina. It is a rod-rich eye but contains two types of cones, short (S) and long wavelength (L) -cones with spectral peaks around 429 and 555 nm, respectively [2]. Together, these cone populations represent around 2%–3% of cells in the periphery of the retina, but >5% in an area centralis temporal to the optic nerve head [3]. A cone-rich visual streak is also present stretching horizontally from the area centralis, and is more marked in dolicocephalic breeds [4]; L cones outnumber S cones by about 10:1 [3]. There is no equivalent of the cone-dominated foveola of humans.

Although PRA in dogs usually manifests as a breed-specific phenotype, instances of different but allelic mutations leading to PRA have appeared independently in related breeds [5,6], or the same PRA mutation may be shared by several breeds. For instance, progressive rod cone degeneration (*prcd*) occurs in 18 dog breeds and breed subtypes [7]. Some breeds, such as the Poodle and the Miniature Schnauzer among several others, appear to express more than one form of PRA, complicating the interpretation of DNA testing results.

Similar phenotypic heterogeneity, as well as extensive genetic heterogeneity, is seen in human retinopathies, such as retinitis pigmentosa (RP) [8,9] and Leber congenital amaurosis (LCA) [10,11]. Inter- and intrafamilial phenotypic variation, including age of onset and disease progression, have been commonly described in human retinal degenerations [12]. To date, 143 genes have been implicated in human inherited retinopathies at RetNet. In humans, DNA testing is possible but is more complicated and larger in scale than in dogs due to the numerous mutations that have to be screened for, the outbred population structure, and the common occurrence of compound heterozygosity [13-15].

Since the 1990s, The MLHD dog has rapidly gained popularity in Japan with more than 100,000 annual Miniature Dachshund registrations to the Japan Kennel Club (JKC) between 2001 and 2006. Consequently, conditions that allow higher incidence of genetic diseases have emerged.

The PRA phenotype in MLHDs has been studied in an inbred research colony. Initially, the disease was examined clinically and histologically by Curtis et al. [16] and was described as an autosomal recessive early-onset form of PRA with all affected cases becoming blind by the time they were 2 years (2y) old. Turney et al. [17] performed an electroretinography (ERG) study that identified an initial reduction of the cone photoreceptor function, which was classified as cone-rod dystrophy 1 (*cord1*). More recently, Lhériteau et al. [18] studied affected MLHDs derived from dogs used in previous studies [16,17,19] to explore potentiality for gene therapy. Their observations agreed with previous descriptions that the PRA condition in MLHDs is an early-onset cone-rod dystrophy reaffirming the need for an early initiation of therapies. It was also identified that the thinning of the retina was caused by apoptotic photoreceptor cell death.

Using the same colony dogs as in previous studies [16,17], Mellersh et al. mapped the *cord1* locus to a 14.15 Mb region on dog chromosome 15 (CFA15), which contained a strong candidate gene: retinitis pigmentosa GTPase regulator-interacting protein 1 (*RPGRIP1*) [19]. A 44 bp insertion of a polyA_29_ tract flanked by a 15 bp duplication (A_29_GGA AGC AAC AGG ATG; *RPGRIP1* insertion) was identified in the presumptive exon 2 of *RPGRIP1*. This insertion causes a frameshift that truncates the gene early in exon 3. The *RPGRIP1* insertion segregated completely with the *cord1* phenotype in that colony.

The RPGRIP1 protein was initially identified through the interaction with RPGR [20,21], which is responsible for an X-linked retinopathy in humans as well as X-linked PRA in Samoyed and Siberian Husky dogs [22]. Although its role in visual function has not been established, RPGRIP1 has been proposed to anchor regulatory complexes at the photoreceptor connecting cilium [21]; it is also thought to be essential for RPGR function [23] and to have functions in disk morphogenesis [24] and in the structure of the ciliary axoneme [25]. Moreover, mutations in *RPGRIP1* have been identified as causing human LCA type 6 [26], cone-rod dystrophy (CRD) [27], and RP [28]. Also, *RPGRIP1* knockout mice show retinal abnormalities [24], an indicator of the importance of RPGRIP1 in visual function. Recently, Wiik et al. identified a mutation in *NPHP4*, truncating a domain known to interact with RPGRIP1 as a cause for cone-rod dystrophy in Standard Wirehaired Dachshunds (SWHDs) [29]. *RPGRIP1* is a common causative gene in human cases of LCA, CRD and RP and clinical phenotypes in the two species closely mimic each other (early-onset for LCA, cone-led photoreceptor degeneration for CRD). Hence *cord1* in MLHDs has been described as an ideal naturally occurring animal model for human treatment and for the understanding of the disease pathology [18]. The use of canine models of human retinal degenerations in therapeutic development has been widespread and successful [30-32].

MLHDs are known to be predisposed to PRA [33], but no comprehensive study has been done in dogs from the pet population to date. We therefore investigated the correlation between the *RPGRIP1* insertion—the mutation that fully correlated with *cord1* in the research colony dogs—and hereditary blindness in pet MLHDs. We also studied other dog breeds since the mutation in exon 2 of *RPGRIP1* was observed recently in the English Springer Spaniel (ESS) by Johnson and coworkers (personal communication, Dr. Gary S. Johnson, College of Veterinary Medicine, University of Missouri, MO). This paper describes substantial variations in phenotype among *RPGRIP1*^−/−^ dogs as well as PRA in cases with other genotypes, suggesting that the genetic etiology of the disease in MLHD and other breeds may be more complex than initially thought.

## Methods

### Animals

#### MLHDs

Samples were collected from three different MLHD sources: 1) Privately owned MLHDs were referred to Veterinary Medical Center (VMC), University of Tokyo either for blindness related to retinopathy (n=64; 0.3–15y) or for nonophthalmologic reasons (n=200; 0.4–13.1y). These dogs were bred and born in Japan and where pedigree certificates were available, it was confirmed that they were unrelated to each other according to a three-generation pedigree issued by the JKC. 2) There were 18 related MLHDs from an extended family (Family K) with a PRA case that was initially presented to VMC. The dogs were born at the same kennel, and had since been housed together in a common environment and with the same diet. The proband was adopted after the initial examination and had been kept separately from his kin during the course of the follow-up examinations for the other family members. 3) The last MLHD source came from nine privately owned MLHDs with PRA whose DNA samples were submitted to the Animal Health Trust (AHT), UK from various countries. These cases were used only for the fine-mapping of the *cord1* region.

#### Non-MLHD breeds

This set consisted of 510 dogs of 66 different breeds which had no obvious visual deficits at sample collection with the exceptions mentioned in the following lines. Dogs from Japan included 78 healthy dogs of different breeds, one Miniature Schnauzer PRA case from the pet population, and 79 laboratory Beagles (mean age±SD: 3.0±2.4y). From the UK, we recruited 278 pet dogs who were referred to the Queen’s Veterinary Hospital, University of Cambridge with nonophthalmologic complaints, four Lhasa Apso PRA cases, and one PRA-affected Newfoundland. Finally, there was a group of 69 pet ESSs whose samples were submitted to the AHT from within and outside of the UK; these ESSs consisted of 15 PRA cases, 28 controls, and 26 dogs of unknown phenotype.

All tests on Japanese dogs were performed either on patients with consent from the owners or on laboratory Beagles kept under the regulations of the Animal Care and Use Committee of Faculty of Agriculture, University of Tokyo. All DNA testing performed in the UK was done on DNA samples collected as surplus from buccal swabs or blood specimens submitted for routine *cord1* tests or clinical biochemistry. All samples were kept anonymous for research purposes after owner consent had been obtained.

### Clinical diagnosis

The phenotype of MLHDs from the Japanese pet population was determined by the same veterinary ophthalmologist (KK); other PRA cases were diagnosed by each referring veterinarian. Nine members of Family K were examined at three occasions, with the follow-ups at 2.8 and 4.2 years after initial examination, and eight other dogs were examined at the last two occasions; the proband was examined once. The age of the dogs at each examination is shown in Table 1.

**Table 1:** Progression of the PRA phenotype in 18 related MLHDs.

**Dog ID**	***RPGR1P1* genotype**	**Sex**	**Age (years)**	**Clinical signs**	**Scotopic ERG**	**Funduscopy**		**Menace reaction**	**PLR**	**Dazzle reflex**
						**Tapetal reflectivity**	**Retinal vessels**			
MLD21	- / -	M	5	Blind (<<5y~)	ND	↑	↓↓	0	1	0
MLD1	- / -	F	6.3	NA	↓	↑ (periphery)	↓ (slight)	2	1	2
			9.2	NA	ND	ND	↓ (periphery, severe)	2	1	1.5
			10.6	Blind	ND	ND	↓	0	1	0
MLD2	- / -	F	5.2	Blind (3y~)	ND	ND	↓↓	0	1	0
			7.9	Blind	ND	ND*	ND*	0	0	0
			9.4	Blind	ND	ND*	ND*	0	0	0
MLD3	- / -	F	3.6	NA	↓	ND**	NA	2	2	2
			6.4	NA	ND	ND**	↓ (periphery, slight)	2	2	2
			7.8	NA	ND	Pigmentation (non-tapetum)	↓	2	1	1
MLD4	- / -	F	2.4	Mydriasis	↓↓↓ (flat)	↑ (perifery)	↓ (periphery)	2	1	1
			5.2	Blind	ND	↑	↓↓	0	1	1
			6.6	Blind	ND	↑	↓↓	0	0	0
MLD5	- / -	M	5.2	NA	↓	↑ (perifery)	↓ (periphery)	2	2	2
			7.9	NA	ND	↑ (slightly)	↓ (periphery, slight)	2	2	2
			9.4	NA	ND	↑	↓	1	1	1
MLD6	- / -	M	3.3	NA	↓	↑ (perifery)	NA	2	2	2
			6	NA	ND	NA	NA	2	2	2
			7.5	NA	ND	Degeneration (periphery)	NA	2	2	2
MLD7	- / -	M	5.2	NA	↓	↑ (perifery)	↓ (periphery)	2	1	1
			7.9	NA	ND	Choroidal vessels visible (periphery)	NA	2	2	2
			9.4	NA	ND	Pigmentation (non-tapetum)	↓	0/1	1	1
MLD9	- / -	M	2.4	Mydriasis (slight)	↓	↑↓ (periphery)	NA	2	1	1
			5.2	NA	ND	Depigmentation (non-tapetum)	↓ (slight)	2	2	2
			6.6	Blind	ND	↑	↓↓	0	0	1
MLD10	- / -	F	0.4	NA	↓	NA	NA	2	2	2
			3.3	NA	ND	NA	NA	2	1	2
			4.7	NA	ND	↑ (slightly)	↓ (periphery)	2	1	2
MLD8	- / -	M	2.8	NA	NA	NA	NA	2	2	2
			4.2	NA	ND	NA	NA	2	2	2
MLD11	- / -	M	2.3	NA	ND	↓ (periphery)	NA	2	2	-
			3.8	NA	ND	Degeneration (periphery)	NA	2	2	2
MLD12	- / -	F	2.8	NA	ND	NA	NA	2	2	2
			4.3	NA	ND	NA	NA	2	2	2
MLD13	+ / -	F	2.2	NA	NA	↑ (disseminated)	NA	2	2	2
			3.7	NA	ND	Abnormal reflection (partially)	NA	2	2	2
MLD14	+ / -	F	1.9	NA	ND	NA	NA	2	2	2
			3.3	NA	ND	NA	NA	2	39845	1
MLD15	+ / +	F	10.1	NA	ND	NA	NA	2	1	2
			11.6	NA	ND	NA	NA	2	1	2
MLD18	+ / +	M	8.3	NA	ND	NA	NA	2	1	2
			9.8	NA	ND	NA	NA	2	0	2
MLD19	+ / +	F	8.3	NA	ND	NA	NA	2	2	2
			9.8	NA	ND	Pigmentation (periphery)	NA	2	1	2

Changes of the retinal status in 18 MLHDs from an extended family (Family K) with shared environment over 4.2 years (n=10) or 1.4 years (n=8) periods are shown. The PRA phenotype was evaluated by owner interview, routine ophthalmologic examination including fundoscopy, and, in 10 dogs, also by electroretinography (ERG). *RPGRIP1* genotypes are denoted as follows: wildtype homozygote (+/+); heterozygote (+/−); insertion homozygote (−/−). Scoring of reaction/reflexes was done using the following scale: not abnormal (2); slightly abnormal (1); abnormal (0). Up arrows (↑) mark tapetum hyperreflective, while down arrows (↓, ↓↓, or ↓↓↓) denote slight, intermediate or severe decrease of ERG response/hyporeflectivity of tapetum/attenuation of retinal vessels. Abbreviations: pupillary light reflex (PLR); not abnormal (NA); not determined (ND); not determined due to hypermature cataract (ND*); not determined due to narrow tapetum region (ND**).

Criteria for the diagnosis of PRA were clinical histories of progressive and not sudden visual impairment, and fundoscopic evidence of bilateral progressive retinal degeneration. Clinical histories concerning the onset and development of behavioral evidence of visual deficit such as bumping into objects, inability to chase moving objects, decreased activity were carefully reviewed and the age of onset was determined as the age when the earliest possible sign of visual deficit was noticed by the owner. A general ophthalmologic examination for visual function included menace response, pupillary light reflex (PLR), and dazzle reflex. Indirect fundoscopy was performed with 14, 20, and 28 diopter lenses (Nikon, Tokyo, Japan) and, in some cases, fundic photographs were taken with a fundus camera (Genesis-D; Kowa, Tokyo, Japan). Slit lamp biomicroscopy and intraocular pressure measurements was also performed to examine other ophthalmologic abnormalities.

Full-field ERG was performed with a custom-built, computer-based ERG acquisition system (ERG for Windows 95 Ver. 1.05, © 1995; Loew Lab at Cornell University) [34] with procedures based on methods described by others [34,35] and modified as follows. Note that PRA in the MLHD was regarded as rod-cone degeneration until the ERG study by Turney et al. [17]. Therefore, MLHDs which underwent ERG at the initial stage of this study were examined only for the scotopic response as part of a routine clinical ERG for MLHDs suspected for PRA, and the photopic ERG procedure was omitted in these subjects. General anesthesia was induced with 6 mg/kg intravenous propofol (Rapinovet®; Schering-Plough Animal Health, Tokyo, Japan) and 0.5 mg/kg rocuronium (Eslax®; Schering-Plough, Tokyo, Japan). After intubation, anesthesia was maintained with constant infusion of 25–30 mg/kg/h propofol, and 1.5 mg/kg/h rocuronium was used to prevent down rotation of the eye. Pupils were dilated with 5 mg/ml tropicamide (Mydrin®P; Santen Pharmaceutical, Osaka, Japan). After 20 min of dark adaptation, each eye was tested separately with stimuli from a white-light LED at four increasing intensity steps, with intervals of four minutes in between each step. The light intensity was increased by 1 log cd/m^2^, and the highest intensity was approximately 18,400 cd/m^2^, as measured with a luminance meter (LS-100; Konica Minolta Sensing, Inc., Osaka, Japan). In most of the MLHDs, a fifth higher intensity stimulus was recorded. The dogs were then light-adapted for 20 min, and photopic ERGs were recorded at 31 Hz with the light intensity of approximately 35,900 cd/m^2^ as measured with a luminance meter. Amplitude of the a-wave was measured from baseline to the peak of the negative deflection, whereas the b-wave amplitude was measured from the peak of the a-wave to the first positive peak of the ERG.

With the 10 MLHDs of Family K, scotopic ERG was performed once at the time of first examination when they showed no apparent visual defect. Both scotopic and photopic ERGs were performed in four laboratory Beagles: two dogs with a longer homozygous insertion at the *RPGRIP1* insertion site (*RPGRIP1*^−L/−L^) and two controls (*RPGRIP1*^+/−L^ and *RPGRIP1*^+/+^). Scotopic ERG with or without photopic ERG was also performed shortly after the onset of PRA in cases which had become blind suddenly and showed no fundoscopic abnormality at presentation. In these cases, sudden acquired retinal degeneration (SARD) was diagnosed when there was undetectable ERG despite only minor or no detectable abnormalities were present at fundoscopy.

### DNA extraction

DNA was extracted from whole blood or buffy coat samples in EDTA or heparin, using the DNeasy Blood (Qiagen, West Sussex, UK) or the EZ1 DSP DNA Blood kits (Qiagen, Tokyo, Japan).

### Genotyping of the *RPGRIP1* insertion

The genotype of the locus containing the insertion in exon 2 of *RPGRIP1* was determined by sizing fluorescently labeled PCR products. The 10 μl PCR reaction contained 4 ng of genomic DNA, 1.5 mM MgCl_2_, 1.5 pmol of a fluorescent-labeled forward primer (Sigma-Proligo, Dorset, UK), and 1.5 pmol of the unlabeled reverse primer (MWG, Ebersberg, Germany), 0.2 mM of each dNTP (Invitrogen, Paisley, UK), and 0.625 U of Taq Polymerase (Invitrogen). Primer sequences are shown in Table 2. PCR amplification was performed with an initial denaturation at 94 °C for 5 min, followed by 12 cycles of touch-down PCR (denaturation at 94 °C, 20 s; annealing at 70 °C, descending 1 °C/cycle to 59 °C for 20 s; and extension at 72 °C for 20 s), and 30 cycles of 94 °C, 20 s; 58 °C for 20 s, and 72 °C for 20 s, with a final extension at 72 °C for 10 min. PCR products were sized by capillary electrophoresis using the CEQ8000 Genetic Analysis System (Beckman Coulter, High Wycombe, UK), and the results were analyzed with the Fragment Analysis software that came with the instrument.

**Table 2:** PCR primers for insertion screening and marker genotyping.

**Feature**	**Sequence type**	**CFA15 location (Mb)**	**Forward primer (5′>3′)**	**Reverse primer (5′>3′)**	**Reference**
*RPGRIP1*_insertion*	Gene	21.34	CTTAAGGAGAACACAAGGTAC	GAAGAGCACATGTTGGTGAAGG	[19]
CAMC15.001	Microsatellite	16.74	TTCGCTTCCTCCCTCACATG	TGAGCTGCAGACAAAGGCC	-
CAMC15.036	Microsatellite	17.32	CCTGTGTGGCAGCAGTTGAAT	ACATTGGGTTCCGCATTCAGT	-
CAMC15.037	Microsatellite	18.13	TTTCCAACCTCCCTCCAACC	TGGCGCGGTGGTTTAGCAT	-
CAMC15.038	Microsatellite	18.76	GCTACATGTCAGGCGTTGTGT	TCCTCGTCTCTACAGTGGGCT	-
CAMC15.006	Microsatellite	19.48	CCCGCACCACATGCTCTC	AGCTACGTCAGCTTCTCTAC	-
CAMC15.039	Microsatellite	19.87	AACGCTTAGCTTGCTTCCACG	CATCATCGGGGAAACCCAAGT	-
CAMC15.040	Microsatellite	20.39	ATGGTGGTAATCACGGTGCAA	TCCCCCATTATTGGATGGCCT	-
CAMC15.009	Microsatellite	20.9	TGGCTCAGCGGTTTATTGCC	ACTTCTGCTGGTTGGACAGG	-
CAMC15.041	Microsatellite	21.05	CAGGTGTGAGTTGTGGGTCTT	AGCTGTTCTGTGGGGTGCTA	-
*RPGRIP1*_intron 2	Gene	21.34	CCTTGTGTTCTCCTTAAGTC	TGAGCTTTGTTTGCCTTTGG	-
CAMC15.029	Microsatellite	21.56	GCCTGGCTAGCTCAGTTGA	AACCCAAAGCTGGGCTTAATC	-
CAMC15.034	Microsatellite	21.89	CATGTGCTTCCCAGTCCTTT	ATAGATGGGGTGCCTGAGTG	-
CAMC15.030	Microsatellite	22.2	AAAGGAGGCCCGGATATTTAT	TGAGCACCCCTTTACCATTC	-
CAMC15.035	Microsatellite	22.24	GAAAAGGAGTACCGCCACC	GCTTGGGGGAAGAAACCTAC	-
CAMC15.031	Microsatellite	22.79	CAGCCAATTGTGGCTAGTGA	TGCCCCAATCTACCTTTCTG	-
CAMC15.013	Microsatellite	23.28	AATGAGTCCTACATCAGGCTC	GGACTCCATGTCAAGGGTTG	-
CAMC15.032	Microsatellite	23.95	TTCTCCCTCTGCCTGTGTC	TCCTGAGTGCTAGGCAGTTT	-
CAMC15.033	Microsatellite	24.45	TCAAGACCCTCAAAGGCATAA	GCACCCCGATGTTTATTGTC	-
CAMC15.016	Microsatellite	25.1	TGCTGATTAGGCTGCTCAAG	AAACCAGCTGCTTGGGACAC	-
CAMC15.019	Microsatellite	26.24	TCTCCTTTGACCCAAATTCCC	AGCACTCAGGTTCAAGAGCC	-
CAMC15.023	Microsatellite	28.35	TGTCTCCTGAGGCATGGGA	TCAGGTCCTCTCCCCATTTT	-
FH3813	Microsatellite	28.91	GATAGAGCTCCGTATCATGCTC	TCTTTGATCAACTACCTCATGG	[46]
CAMC15.027	Microsatellite	30.25	TGAACATAGGGGAAGGGAGG	TCCCCTCCAGCAGTATGCA	-

*RPGRIP1* primers used to identify the presence of the insertion allele (*) and primers for polymorphic markers used for genotyping of the initially mapped *cord1* locus on CFA15 are shown. Positions correspond to CanFam2.0 (accessed Oct 2008).

### Sequencing of the *RPGRIP1* insertion site

The region spanning the *RPGRIP1* insertion site was amplified by touch-down PCR as described except that the primers were unlabeled. PCR products were purified with the QIAquick PCR purification kit (Qiagen, West Sussex, UK) and then sequenced bidirectionally with the GenomeLab DTCS-Quick Start Kit (Beckman Coulter, High Wycombe, UK) following the manufacturer’s protocol. Sequencing products were run and analyzed on a CEQ8000 Genetic Analysis System.

### Microsatellite typing

Fine-mapping of the *cord1* critical region on CFA15 (16.54–30.68 Mb; coordinates as in CanFam2.0) was performed by identifying microsatellite markers by analyzing the appropriate canine contig sequences with Tandem Repeat Finder [36], followed by primer design around those microsatellites using Primer3 [37]. The list of primers for these microsatellites is presented in Table 2. Microsatellites were sized with a fluorescently labeled forward primer and an unlabeled reverse primer, and amplified with touch-down PCR. Genotypes were determined by sizing the products on a CEQ8000 Genetic Analysis System.

### Association analysis

The distribution of microsatellite allele frequencies in the PRA cases was compared against the distribution in the controls aged 4 or older by using the T2 chi-square test in the program CLUMP [38]. Since contingency tables for microsatellite data may be sparsely populated, the T2 test collapses the columns of those alleles with low frequencies, thus preventing the distortion of computed chi square values. The level of significance was estimated by computing the chi-square value of 10^11^ randomly generated contingency tables having the same conditional marginals as each one of the input tables.

The T2 test was also used to compare the distribution of *RPGRIP1* alleles between controls of MLHD examined before 4y and those examined at 4y or later. At the marker density used here, genome-wide significance was achieved in the dog at p<5×10^−6^ (Sidak test) [39].

## Results

### Variability of the onset of visual impairment in sporadic PRA cases

**Figure 1:**
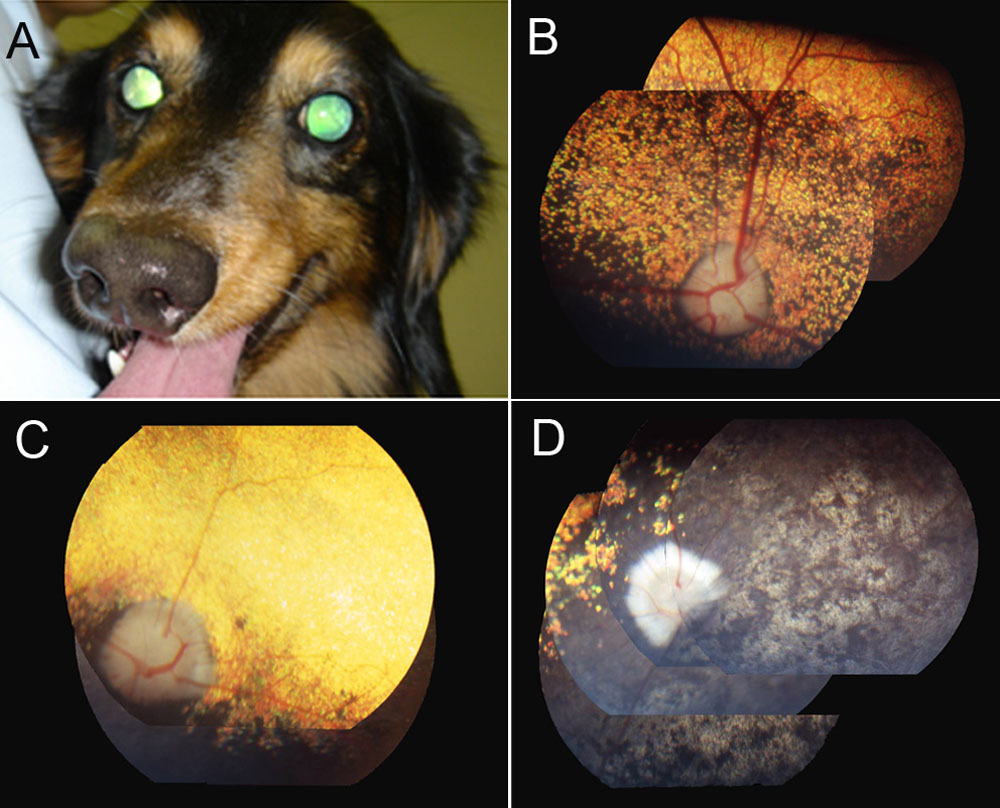
Facial and fundoscopic characteristics of PRA. **A**: Typical PRA-affected cases show mydriasis and increased reflection from the fundus. **B**-**D**: MLHD fundus photographs of a control without visual dysfunction (**B**, 5y), moderately (**C**, 3.4y) and severely (**D**, 5y) affected PRA cases with blindness. Note the tapetal hyperreflectivity (**C**), the attenuation of the retinal vessels, the pale optic disk, and the pigmentation of the nontapetum (**D**).

Owners of 64 unrelated MLHD with sporadic retinopathy from the Japanese pet population were interviewed, to review clinical histories of the onset of visual deficit. These dogs were also given ophthalmologic examinations (Figure 1).

**Figure 2:**
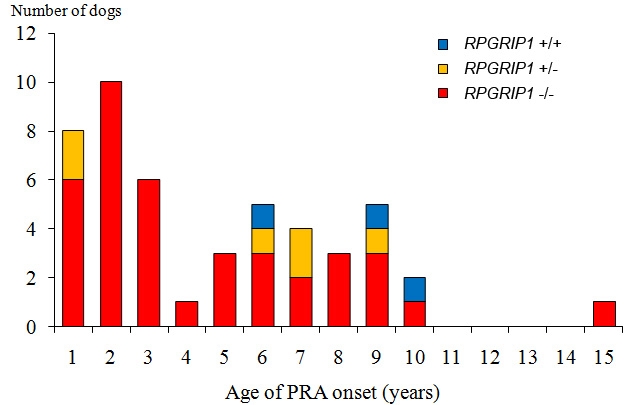
Distribution of the age of PRA onset in sporadic MLHD cases. Presented are 48 sporadic PRA cases of MLHDs from the Japanese pet population. These are shown according to the age of onset. The dogs were already blind at the time of presentation and the age of onset was determined by the earliest possible sign of visual impairment noticed by the owner. An additional 11 sporadic PRA cases from Japan, used for mapping purposes, are not shown here since the information on the age of onset was uncertain or unknown. *RPGRIP1* genotypes are denoted as follows: wildtype homozygote (+/+); heterozygote (+/−); insertion homozygote (−/−).

Of these 64 cases, 59 were PRA and 5 were SARD, an acquired retinopathy. Of the PRA cases, the age of onset could not be determined for 11 cases due to ambiguity or lack of information. For the rest of the cases (n=48) the age of onset could be identified. Half of the cases appeared before 4y with the highest peak at 2y, while the other half were spread between the ages of 4y and 10y with a single outlier with an age of onset of 15y (Figure 2). This last individual showed no fundoscopic abnormality at a previous examination aged 10y.

### Phenotypic variety in an extended MLHD family

**Figure 3:**
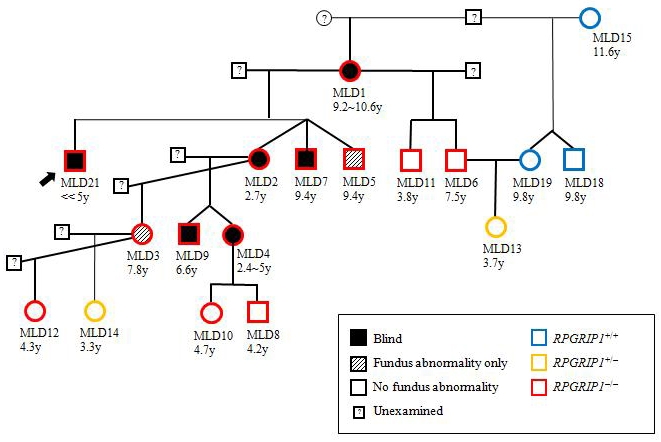
Pedigree of an extended family of MLHDs with shared environment. The family tree of Family K shows the proband (arrow), the dog’s three siblings from another pregnancy, and 14 other related members for which the retinal phenotype was examined over a period of 1.4 to 4.2 years. In the PRA affected cases, the age (y, years) under each symbol indicates the age of onset of behavioral (bold symbols) or fundoscopic (hatched symbols) abnormalities. In the dogs with no apparent visual dysfunction, the age corresponds to that at the last fundoscopic examination. Some members of the family were not examined in this study and are omitted from the pedigree. The following symbols are used: square, male; circle, female; bold, blind; hatched, abnormal fundus with no apparent visual dysfunction; white, no fundoscopic abnormality with no apparent visual dysfunction. *RPGRIP1* genotypes are denoted as follows: wildtype homozygote, in blue (+/+); heterozygote, in yellow (+/−); insertion homozygote, in red (−/−).

Eighteen members of an extended MLHD family (Family K) including the proband affected with PRA (dog MLD21) were examined for the PRA phenotype (Figure 3, Table 1). Nine dogs were examined on three occasions, subsequent examinations taking place 2.8y and 4.2y after the first examination. The other nine dogs were examined on the last two occasions only. The proband had been raised separately from the other dogs since the diagnosis of PRA, and phenotype from a single examination is shown. Only the proband and one other sibling (MLD2) initially appeared to have impaired vision as examined by behavior, menace response, PLR, and dazzle reflex. Four other members of this family developed visual impairment during the course of the study. The other 12 dogs showed no apparent visual dysfunction.

**Figure 4:**
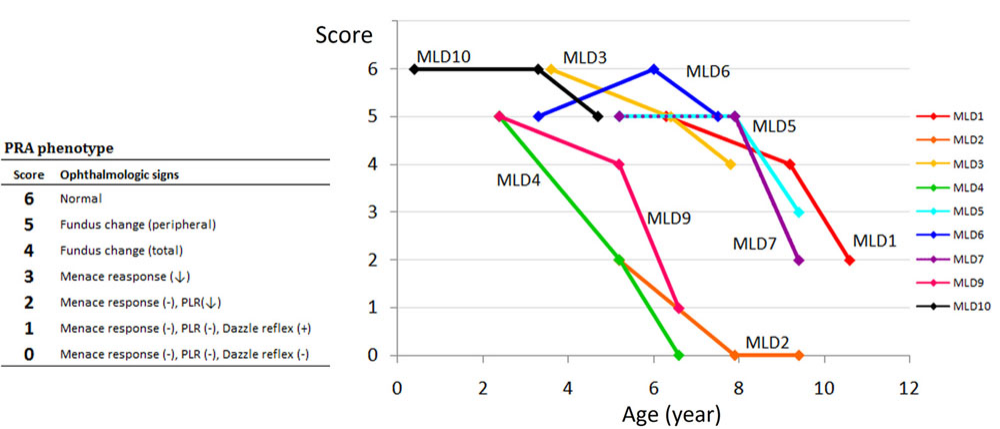
Progression of the PRA phenotype in nine *RPGRIP1*^−/−^ MLHDs an extended family with shared environment. The nine dogs shown are all *RPGRIP1*^−/−^ with shared environment and were examined three times over a 4.2 year period. PRA phenotypes over a 4.2-year period are scored according to ophthalmologic abnormalities determined by fundoscopy, menace response, PLR, and dazzle reflex. A decreasing score indicates disease progression. Animals with scores below 3 are functionally blind and were included in the PRA affected group in the association analysis. MLD2, 5, and 7 and MLD4 and 9 are siblings.

All the dogs were housed together and shared the same environmental conditions, but within the family, even between sibs, there were marked differences noted in the age of onset and disease progression (Figure 4, Table 1). MLD2, 5, and 7 were siblings, but the onset of visual deficit appeared much earlier in MLD2 at 2.7y, while MLD7 showed no visual dysfunction at 7.9y, becoming blind at 9.4y; the third dog (MLD5) retained eyesight throughout the study (last examined at 9.4y), although fundus abnormality (hyperreflectivity and retinal vessel attenuation) developed gradually, starting at 7.9y.

**Figure 5:**
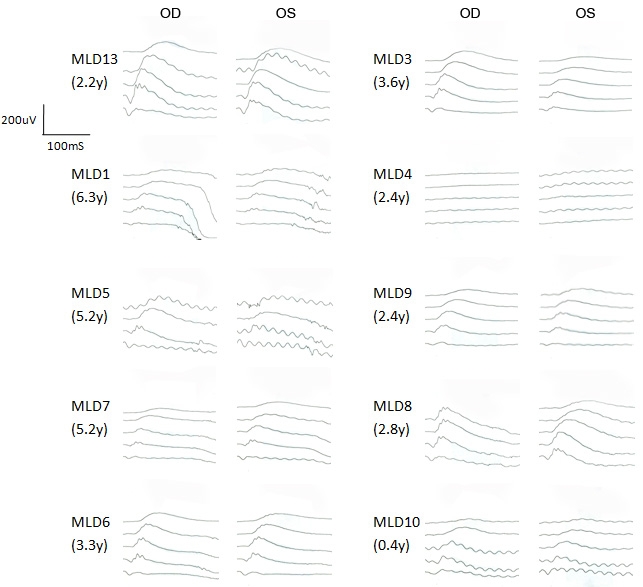
Scotopic ERG in related MLHDs with no obvious sign of visual impairment. Scotopic ERG intensity series of 10 MLHDs from Family K at initial examination is shown. ERG recordings show scotopic responses with five increasing light intensity from top to bottom differing by 1 log cd/m^2^. The age in years (y) corresponds to the age when ERG was performed; at that time, none of the dogs showed apparent visual deficit. All dogs represented are *RPGRIP1*^−/−^, except MLD13 (*RPGRIP1*^+/−^). The data of the highest light intensity was not recorded in MLD5 or in the right eye (OD) of MLD8. OS indicates left eye.

Scotopic ERG was performed in 10 of these dogs at their initial presentation (Figure 5). None of these dogs showed apparent deficit of visual function as examined by behavior, menace response, PLR, and dazzle reflex (Table 1). A moderate (>25%) to marked reduction of ERG response (a- and b-waves) was observed in all of the *RPGRIP1*^−/−^ MLHDs when compared with an *RPGRIP1*^+/−^ dog (MLD13); note that the reduction was not always proportionate to the age of the dog tested. In particular, the ERG response was almost undetectable in MLD4 and was also severely reduced in MLD1, 7, 9, and 10. Thus, by ERG criteria, all *RPGRIP1*^−/−^ dogs tested had some degree of retinal function deficit, although they retained sufficient vision to appear “normal” to the owner.

### Genotype-phenotype discordance in the MLHD pet population

For this study, we screened 59 sporadic PRA cases, five SARD cases, and 200 controls of MLHDs from the Japanese pet population for the insertion in exon 2 of *RPGRIP1*; this insertion has previously been associated with PRA (*cord1*) in this breed [19]. Several discordances between the clinical phenotype and the genotype were observed. Among 200 randomly sampled control MLHDs without apparent clinical signs, 32 dogs (16.0%) were *RPGRIP1*^−/−^, while 12 out of 59 PRA cases (20.3%) were non-*RPGRIP1*^−/−^ (Table 3). All except two of 24 PRA cases with known onset before 4y were *RPGRIP1*^−/−^ (Figure 2). The two exceptions were *RPGRIP1*^+/−^ and, perhaps surprisingly, they had the earliest ages of onset among all PRA cases, at 3 and 4 months of age. Of 24 PRA cases with known onset at or after 4y, seven were non-*RPGRIP1*^−/−^ (four *RPGRIP1*^+/−^ and three *RPGRIP1*^+/+^). Among the 200 control dogs, there was no significant difference in the distribution of the *RPGRIP1* insertion alleles when comparing 60 dogs under 4y against 140 older dogs (p=0.507, chi-square test; Table 4).

**Table 3:** Genotype-phenotype- correlation in MLHDs of sporadic retinopathy cases and controls.

**Phenotype**	***RPGRIP1* genotype**			**Total number of dogs**
**+/+**	**+/−**	**−/−**
Control	81 (41%)	87 (44%)	32 (16%)	200
PRA	6 (10%)	6 (10%)	47 (80%)	59
SARD	4 (80%)	0 (0%)	1 (20%)	5
Total	89(33%)	93 (35%)	82 (33%)	264

Correlation of the retinal phenotype and the *RPGRIP1* insertion genotype were studied in dogs of the Japanese MLHD pet population. Sporadic cases of PRA and sudden acquired retinal degeneration (SARD), and controls with no apparent visual dysfunction showed substantial discordance seen in controls dog with *RGPRIP1^−/−^* and PRA cases that were not *RPGRIP1^−/−^*. *RPGRIP1* genotypes are denoted as follows: wildtype homozygote (+/+); heterozygote (+/−); insertion homozygote (−/−).

**Table 4:** *RPGRIP1* genotype of control MLHDs from different age groups.

**Age at examination (years)**	***RPGRIP1* genotype**			**Total number of dogs**
**+/+**	**+/−**	**−/−**
<4	28 (47%)	24 (40%)	8 (13%)	60
≥4	53 (38%)	63 (45%)	24 (17%)	140
Total	81(41%)	87 (44%)	32 (16%)	200

*RPGRIP1* genotypes in control MLHDs without apparent visual dysfunction were compared between two age groups. those younger than 4 years, and those 4 years or older at clinical examination. There was no significant difference of the distribution of the *RPGRIP1* genotypes between the two age groups. *RPGRIP1* genotypes are denoted as follows: wildtype homozygote (+/+); heterozygote (+/−); insertion homozygote (−/−).

Of the 18 members of Family K, 13 dogs were *RPGRIP1*^−/−^, two were *RPGRIP1*^+/−^, and three were *RPGRIP1*^+/+^ (Figure 3, Table 1). Of the *RPGRIP1*^−/−^ dogs, two had become blind by the beginning of the study (one at 2.7y and another, much earlier than 5y), while the other dogs initially showed no apparent sign of visual dysfunction according to their behavior, menace response, PLR, and dazzle reflex. All *RPGRIP1*^−/−^ dogs became blind or expressed typical fundoscopic abnormalities at some point of the study except MLD8 and 12 (4.2y and 4.3y at last examination). However, scotopic ERG of dog MLD8 had shown 40% reduction in a-wave and 25% reduction in b-wave at 2.8y compared to a *RPGRIP1*^+/−^ dog (MLD13; Figure 5). Of the nine *RPGRIP1*^−/−^ dogs examined by scotopic ERG, eight showed reduced response while one case had no detectable response (MLD4). Dog MLD13 (*RPGRIP1^+/^*^−^), the only non-*RPGRIP1*^−/−^ dog studied by ERG, showed no abnormality in the scotopic response despite evidence of a slight fundoscopic abnormality in the tapetal region. The other four non-*RPGRIP1*^−/−^ dogs (MLD14, 15, 18, and 19) showed no clinical signs of retinal degeneration.

### Four other breeds carry the insertion in *RPGRIP1*

To determine whether the insertion in exon 2 of *RPGRIP1* was present in other breeds, we studied 510 dogs from 66 breeds (Table 5). No eye problems were apparent at sample collection in 489 of these dogs. Fifteen out of 69 ESSs, four Lhasa Apsos, one Newfoundland and one Miniature Schnauzer had PRA. The insertion was observed in four additional breeds. In three French Bulldogs and in one Labrador Retriever the insertion was present in heterozygous state. In 15 ESSs affected with PRA, a third of the cases were *RPGRIP1*^−/−^, four were *RPGRIP1^+/^*^−^, and six were *RPGRIP1^+/+^* (Table 6). Of the 28 control ESSs, two were *RPGRIP1*^−/−^, while the majority were *RPGRIP1*^+/−^. The age at examination for these samples was unavailable.

**Table 5:** Screening of the *RPGRIP1* insertion in non-MLHD breeds.

**Breed**	***RPGR1P1* genotype**			**Breed**	***RPGR1P1* genotype**		
	**+/+**	**+/−**	**−/−**		**+/+**	**+/−**	**−/−**
Beagle	41	32 (+/−L)	6 (-L/-L)	Italian Greyhound	1	0	0
English Springer Spaniel	27	35	7	Jack Russel Terrier	9	0	0
Labrador Retriever	15	1	0	Kooiker Hondje	1	0	0
French Bulldog	9	3	0	Lapponian Herder	8	0	0
Akita	1	0	0	Leonberger	1	0	0
Alaskan Malamute	1	0	0	Lhasa Apso	18	0	0
Australian Cattle Dog	1	0	0	Lurcher	7	0	0
Basset Hound	8	0	0	Maltese	2	0	0
Belgian Shepherd Malinois	1	0	0	Miniature Pinscher	1	0	0
Bernese Mountain Dog	7	0	0	Miniature Schnauzer	8	0	0
Bichon Frise	8	0	0	Newfoundland	8	0	0
Border Collie	7	0	0	New Zealand Hantaway	1	0	0
Borzoi	1	0	0	Pomeranian	1	0	0
Boxer	9	0	0	Poodle	7	0	0
Bull Terrier	1	0	0	Pug	3	0	0
Cairn Terrier	6	0	0	Rhodesian Ridgeback	8	0	0
Cardigan Welsh Corgi	8	0	0	Rottweiler	5	0	0
Cavalier King Charles Spaniel	11	0	0	Samoyed	1	0	0
Chihuahua	1	0	0	Schipperke	1	0	0
Cocker Spaniel	10	0	0	Shetland Sheep Dog	2	0	0
Collie Smooth/Rough	8	0	0	Shiba Inu	1	0	0
Dachshund	7	0	0	Shih Tzu	8	0	0
Dalmatian	7	0	0	Siberian Husky	1	0	0
Doberman	7	0	0	Saint Bernard	1	0	0
Finnish Lapphund	6	0	0	Staffordshire Bull Terrier	10	0	0
Flatcoated Retriever	9	0	0	Swedish Lapphund	7	0	0
German Shepherd Dog	6	0	0	Toy Poodle	2	0	0
Golden Retreiver	16	0	0	Weimaraner	8	0	0
Great Dane	8	0	0	Welsh Corgi Pembroke	2	0	0
Great Pyrenees	1	0	0	West Highland White Terrier	7	0	0
Greyhound	8	0	0	Whippet	7	0	0
Hungarian Vizsla	7	0	0	Wire Fox Terrier	1	0	0
Irish Setter	8	0	0	Yorkshire Terrier	7	0	0
Total	426	71	13				

**Figure 6:**
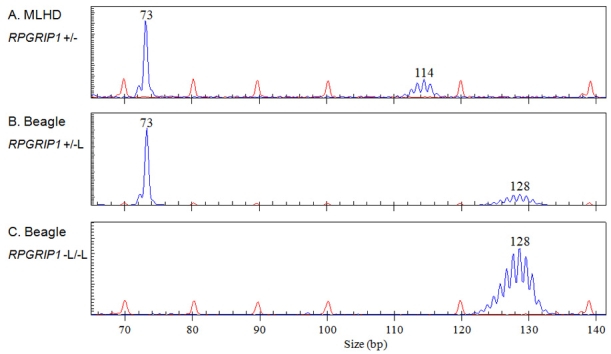
PCR products containing the *RPGRIP1* insertion were sized by capillary electrophoresis in the MLHD and the Beagle. Fragment sizing, by capillary electrophoresis of PCR products containing the *RPGRIP1* insertion in a MLHD (**A**, *RPGRIP1*^+/−^) and two Beagles (**B**, *RPGRIP1*^+/−L^; **C**, *RPGRIP1*^−L/−L^). The 73 bp single blue peak corresponds to the wildtype allele (+), while the blue peaks centered on 114 bp and 128 bp represent the alleles with the insertion (- and -L); these two insertion alleles differ only in the number of adenines in the homopolymeric sequence. The red peaks correspond to the size standard.

Of the 66 dog breeds screened, the insertion was observed in the four breeds shown at the top. *RPGRIP1* genotypes are denoted as follows: wildtype homozygote (+/+); heterozygote (+/−); insertion homozygote (−/−). The insertion allele (-L) in the Beagle had a longer polyA tract than the allele in the other breeds (see Figure 6). Of the four breeds with the insertion, homozygous individuals were found only among Beagles and English Springer Spaniels. Beagles that were *RPGRIP1*^-L/-L^ showed no apparent visual dysfunction, but photopic ERG was undetectable. Some of the English Springer Spaniels were PRA-affected, and the three possible genotypes were found among these cases.

**Table 6:** Genotype-phenotype correlation in English Springer Spaniels.

**Phenotype**	***RPGRIP1* genotype**			**Total number of dogs**
**+/+**	**+/−**	**−/−**
Control	9 (32%)	17 (61%)	2 (7%)	28
PRA	6 (40%)	4 (27%)	5 (33%)	15
Total	15 (35%)	21 (49%)	7 (16%)	43

The *RPGRIP1* insertion genotype was studied in PRA cases and apparently normal controls of the English Springer Spaniel. Although the proportion of *RGPRIP1*^−/−^ dogs were higher in PRA cases, there was marked genotype-phenotype discordance as seen in *RGPRIP1*^−/−^ controls and non-*RGPRIP1*^−/−^ PRA cases. *RPGRIP1* genotypes are denoted as follows: wildtype homozygote (+/+); heterozygote (+/−); insertion homozygote (−/−).

In the Beagles, the fourth breed in which the insertion was found, 79 laboratory animals with no apparent clinical signs were studied. A longer variant of the insertion was observed in the homozygous state (*RPGRIP1*^−L/−L^) in six dogs (7.6%) and in the heterozygous state (*RPGRIP1*^+/−L^) in 32 dogs (40.5%). Overall, this allele had a frequency of 0.28 in the Beagle.

The insertion in ESSs, French Bulldogs, and in the Labrador Retriever was the same length as that in MLHDs, as determined by capillary electrophoresis of PCR products containing it (data not shown). The MLHD insertion contains a homopolymeric sequence of 29 adenines [19]. In the Beagles, however, the polyA tract was found to be approximately 15 bp longer as determined by sizing the PCR products (Figure 6) and sequencing them. The exact length of the polyA tract could not be determined due to slippage during the PCR reaction that generated a heterogeneous population of molecules. However, its length was deduced by comparing the size of the major products in Beagles and in other breeds. The duplicated sequences immediately flanking the polyA tract were identical to those reported previously for the MLHD in all the breeds with insertion.

### *RPGRIP1*^-L/-L^ Beagles showed reduced ERG without apparent visual deficits

**Figure 7:**
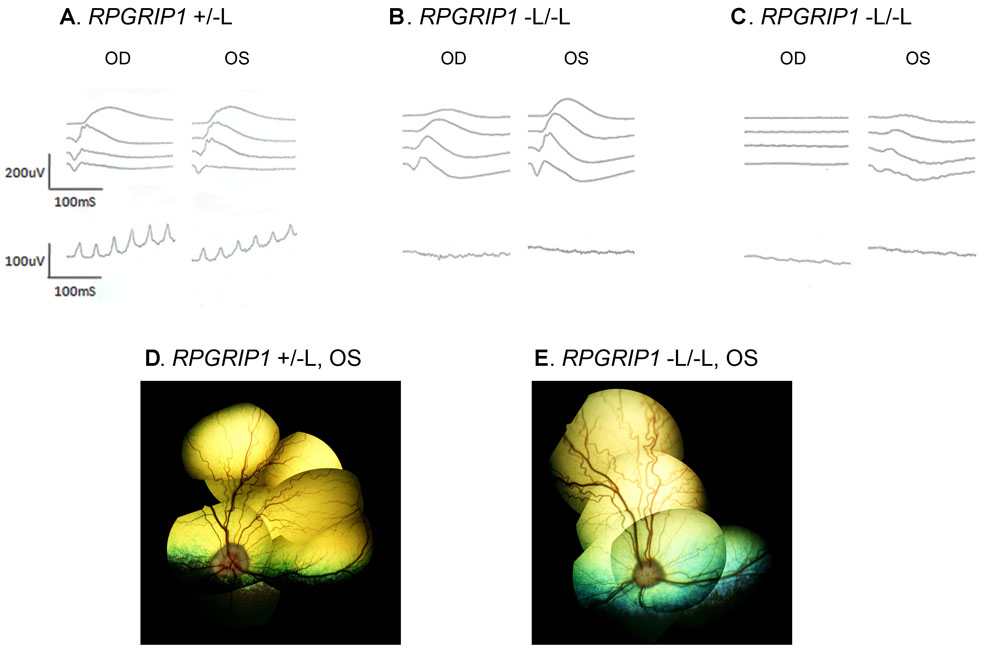
ERG responses and fundus photograph of Beagles with the *RPGRIP1* insertion variant. Bilateral scotopic (top) and photopic (middle) ERG recordings and fundus photograph of the left eye (OS; bottom) of three Beagles: a 5y *RPGRIP1*^+/−L^ dog (**A** and **D**), a 2.8y *RPGRIP*^−L/−L^ (**B** and **E**), and another 2.8y *RPGRIP1*^−L/−L^ dog (**C**). Scotopic responses to a series of light stimuli are displayed with increasing light intensity from top to bottom differing by 1 log cd/m^2^ up to 18,400 cd/m^2^. The photopic response was recorded with 31 Hz flicker stimuli of 35,900 cd/m^2^. Note the apparently normal fundus appearance in the *RPGRIP1*^−L/−L^ dog (**E**) with undetectable cone response (**B**). OD indicates right eye.

As part of an ophthalmologic examination, fundoscopy and both scotopic and photopic ERGs were performed in four Beagles: two *RPGRIP1*^−L/−L^ dogs (both aged 2.8y) and two control Beagles (*RPGRIP1*^+/−L^ and *RPGRIP1*^+/+^, both aged 5y). All four dogs had no visual dysfunction as determined by maze test in light and in dim light, as well as by menace response, PLR, and dazzle reflex. Indirect fundoscopy of the four dogs indicated a slight but not obvious attenuation of the retinal vessels in the *RPGRIP1*^−L/−L^ dogs (Figure 7). With ERG, cone response was undetectable in both *RPGRIP1*^−L/−L^ dogs; one dog showed nearly normal rod response with clear reduction in the right eye only, while the other dog showed no rod response in the right eye and severely reduced response in the left.

### Fine mapping showed the highest association with the disease at the *RPGRIP1* insertion

**Figure 8:**
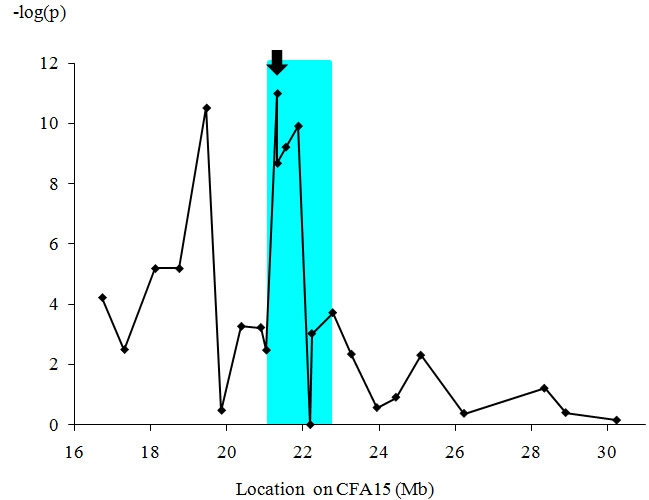
Fine mapping of the *cord1* critical region. Twenty-four polymorphic markers on CFA15 were studied for the association with PRA in MLHDs. In total, 74 PRA cases and 86 controls were used for this fine mapping across the originally reported region of homozygosity [19]. Note that for the marker with the highest value at 21.34 Mb (*RPGRIP1* insertion, arrow) the p-value is below the minimum detectable in the simulation, that is <1x10^−11^. The aqua-highlighted area corresponds to the 1.74 Mb region of homozygosity shared by all PRA cases having two copies of the *RPGRIP1* insertion.

A previous study with an inbred research colony mapped the *cord1* locus to CFA15 and determined a region of homozygosity shared by all the cases and spanning 14.15 Mb [19]. Here, we further mapped this region with 24 polymorphic markers, using 74 PRA cases (59 sporadic Japanese cases, six cases from Family K, plus nine cases from other countries) and 86 controls aged >4y from the MLHD pet population. For each marker, allele distributions were compared between cases and controls. The strongest evidence of association (p<1x10^−11^) was observed with the *RPGRIP1* insertion itself (21.34 Mb) followed by the association with loci at 19.48 Mb (p=3×10^−11^) on CFA15 (Figure 8).

### The *cord1* critical region was reduced to 1.74 Mb

**Figure 9:**
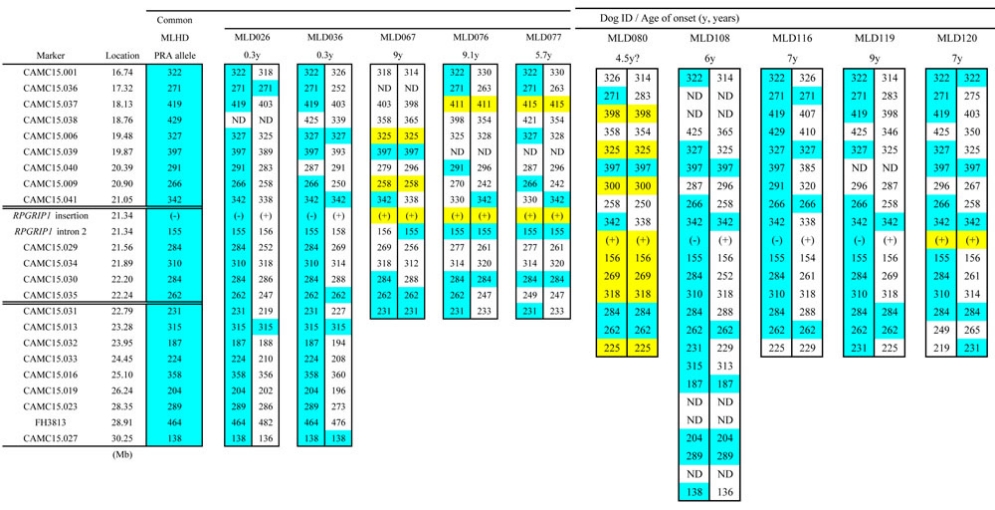
Marker analysis in PRA-affected non-*RPGRIP1*^−/−^ MLHDs. Haplotypes of the *cord1* critical region on CFA15 were studied in 12 PRA-affected non *RPGRIP1*^−/−^ MLHDs. The “common allele” refers to the combination of the most frequent allele observed in the *RPGRIP1*^−/−^ PRA cases (47 sporadic cases and six cases from Family K). For each cell representing an allele in the dog studied, blue shading highlights genotypes identical to the haplotype associated with the *cord1* insertion; yellow highlights alleles that are homozygous in the dog under study but differ from the common allele. The symbol (-) and (+) for the marker *RPGRIP1* insertion each represents the *RPGRIP1* insertion allele and the wildtype allele, respectively. ND indicates that the genotype was not determined.

**Figure 10:**
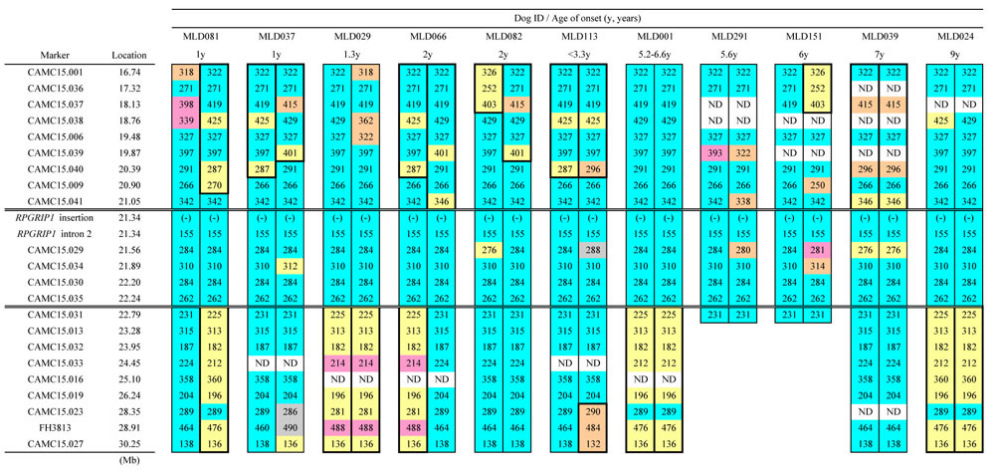
Marker analysis in PRA affected *RPGRIP1*^−/−^ MLHDs. Polymorphic markers in the *cord1* critical region on CFA15 were analyzed in PRA affected *RPGRIP1*^−/−^ MLHDs. The 12 dogs shown are representative of the haplotypes of 47 sporadic cases and six cases from Family K studied. Alleles shared by the majority of the dogs are highlighted in blue (referred to as “common allele” in Figure 9 and Figure 11) and other highlighted colors mark less common alleles. Variants in CAMC15.029 and 034 could be the result of mutation events, rather than recombination since the markers flanking them are the “common alleles.” Therefore, the region of homozygosity was delimited by markers CAMC15.041 and CAMC15.031. The symbol (-) for the marker *RPGRIP1* insertion represents the *RPGRIP1* insertion allele. ND indicates that the genotype was not determined.

Of the 74 PRA cases, 12 Japanese MLHDs were non-*RPGRIP1*^−/−^. Onset age was known in only nine of these (Figure 2). Among these 12 cases, no marker within the published *cord1* critical region [19] was shared as homozygous (Figure 9). Since PRA of some or all of these 12 cases might be associated with loci other than the *cord1* locus, they were excluded. Analyzing the genotypes of the rest of the 62 PRA cases, a region of homozygosity was identified between 21.05 and 22.79 Mb (Figure 10). This 1.74 Mb region contains *RPGRIP1* in addition to 24 known genes and eight predicted ones (CanFam 2.0). None of these genes, apart from *RPGRIP1*, has been implicated in retinopathies in humans, dogs, or mice. Two markers, at 21.56 and 21.89 Mb, did show some variation in *RPGRIP1*^−/−^ dogs. The marker at 21.56 Mb is particularly variable with 17 alleles across the whole MLHD population (74 PRA cases and 86 controls) tested in this work, while that at 21.89 Mb also shows a high level of polymorphism. It is possible that in both cases new alleles have been generated since the entry of the PRA-causative mutation into the MLHD population.

### The haplotype shared between MLHDs and Beagles around the *RPGRIP1* insertion is limited

**Figure 11:**
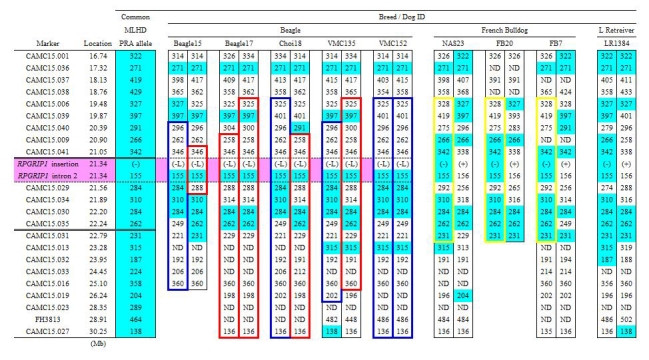
Marker analysis in non-MLHD breeds with the *RPGRIP1* insertion. Polymorphic markers in the *cord1* critical region on CFA15 were analyzed in five *RPGRIP1*^−L/−L^ Beagles, and in four other *RPGRIP1^+^*^/−^ dogs (three French Bulldogs and a Labrador Retriever). The common allele denotes the combination of the most frequent allele observed in *RPGRIP1*^−/−^ PRA cases of MLHDs (47 sporadic cases and six cases from Family K). Blue highlights mark genotypes identical to the common allele in the PRA-affected MLHDs. Haplotype blocks outlined in blue and red (Beagle) and yellow (French Bulldog) represent each breed-specific haplotype. Without taking into account the variation of the insertion length in *RPGRIP1*, the region of homozygosity shared between PRA-affected MLHDs and beagles both homozygous for an insertion in exon 2 of *RPGRIP1* (*RPGRIP1*^−/−^ and *RPGRIP1*^−L/−L^) was delimited by markers CAMC15.041 and CAMC15.029, which is highlighted in pink. The symbol (-) and (-L) for the marker *RPGRIP1* insertion each represents the *RPGRIP1* insertion allele and its variant while (+) represents the wildtype allele. ND indicates that the genotype was not determined.

The aforementioned 24 markers were also studied in non-MLHD breeds carrying the *RPGRIP1* insertion. Four *RPGRIP1*^+/−^ dogs (three French Bulldogs and a Labrador Retriever), and five *RPGRIP1*^−L/−L^ Beagles were analyzed (Figure 11). The shared region adjacent to *RPGRIP1* spanned 3.56 Mb in the French Bulldog and 4.06 Mb in the Labrador Retriever. In the Beagle, disregarding the difference of the insertion length in *RPGRIP1*, only a single distal marker was shared with *RPGRIP1*^−/−^ MLHDs. If the ERG abnormalities in Beagles have the same cause as *cord1* mapped in MLHDs, then this result suggests a maximum 0.51 Mb region containing the mutation causing the disorder, bracketed by markers CAMC15.041 (21.05 Mb) and CAMC15.029 (21.56 Mb) and containing 14 genes (11 known and three unknown) other than *RPGRIP1*.

## Discussion

In previous studies, PRA in MLHDs has been described as a relatively early onset retinal degeneration with ophthalmologic abnormalities being detectable as early as six weeks of age by ERG and 25 weeks by fundoscopy, leading to total blindness by 2y in all the affected MLHDs [16-19,33]. However, those observations corresponded to MLHDs from an inbred research colony. It was not known whether the description reflected the conditions in the general pet population. We therefore studied PRA cases and controls from the Japanese pet MLHD population which the recent increase in popularity has created a large population with many PRA cases. MLHDs from the UK pet population which contributed the founders of the research colony [16,17,19] were also studied.

Contrary to previous studies with the MLHD colony with *cord1*, extensive phenotypic variation was observed in the MLHD pet population. In our study among 59 MLHDs with sporadic PRA, the age at which visual deficit was first noticed varied extensively. Factors such as the amount of light exposure of the dogs, their diet, the extent of familiarity with the living environment, as well as the extent of observation could affect the owner’s perception of the age of onset of visual deficit in these privately owned dogs. Yet a more objective comparison was obtained from the observation of 18 MLHDs from an extended family (Family K) housed together. Ophthalmologic examination in these dogs over a 4.2-year period identified a variable onset of noticeable visual deficit and differential rate of retinal degeneration even between siblings. The group of early-onset dogs with onset around 2y appears to correspond to the phenotype of the MLHD from the research colony previously described [16]. The rest of these dogs showed later onset, after 4y, and with a broad range not seen in the colony MLHDs. Such phenotypic variety is typical in human retinopathies caused by the same mutation on a heterogeneous genetic background [12]. In the dog, on the contrary, most forms of PRA, in which a mutated locus is considered causal, express a relatively uniform phenotype within any given breed due to the relatively homogeneous genetic background of most breeds.

Although there was complete genotype-phenotype correlation in the research colony studied by Mellersh et al. [19], we have observed substantial discordance in the pet population. The fact that *RPGRIP1*^−/−^ dogs with functional vision constitute a high proportion of MLHDs and of ESSs (16.0 and 7.1% of each control group) indicates that this insertion is not by itself sufficient to cause early-onset PRA. In this study, all *RPGRIP1*^−L/−L^ Beagles also retained sufficient vision as to appear normal in standard clinical assessment.

A possible explanation for the lack of complete correlation between the clinical status and the *RPGRIP1* insertion could be that a mutation associated with PRA in MLHDs is located elsewhere in the *cord1* critical region. However, no marker from this region was found to be homozygous in all the PRA-affected MLHDs. We have narrowed the *cord1* region to a 1.74 Mb region containing *RPGRIP1* and 32 other genes. In this region, the strongest evidence of association with PRA was observed with the *RPGRIP1* insertion itself (p<1x10^−11^; 21.34 Mb) followed by the loci at 19.48 Mb (p=3×10^−11^) on CFA15. This gives strong genome-wide significance in support of this region as causative for PRA. Moreover, if the ERG abnormalities in Beagles have the same cause as *cord1* in MLHDs, the region could be further narrowed to 0.51 Mb and contain 14 genes (11 known and three unknown) other than *RPGRIP1*. Given its role in human and mouse retinopathies, *RPGRIP1* remains a strong positional and functional candidate gene, and the insertion in exon 2 is so far the most likely mutation associated with PRA in MLHDs. We argue here that the discordant clinical results are consistent with incomplete penetrance of the insertion mutation, as measured by standard fundoscopic and behavioral tests. Given the variable ages of onset, and that there are dogs with PRA onsets at later life, it follows that the disease penetrance will be more incomplete in younger dogs than older ones. In our sample, of those *RPGRIP1*^−/−^ dogs that do show fundoscopic or behavioral signs, over 50% manifest these before the age of three years (Figure 2), but thereafter there is a gradual increase in the numbers showing these signs over the remainder of the dogs’ lifetimes. We do not have an accurate age structure for the MLHD pet population, as our collection comes from clinics and is likely to be biased toward young and old animals and away from normal “middle-aged” adults. However, based on the animals detailed in Table 3 and Table 4,it appears that a substantial proportion of homozygous *RPGRIP*1^−/−^ dogs do not show fundosopic or behavioral changes from this source in their lifetimes.

In the current study, ERG has proved useful for detecting the earliest sign of retinal degeneration before the appearance of any fundoscopic or behavioral changes. All the *RPGRIP1*^−/−^ MLHDs in which scotopic ERG was measured showed reductions in the rod response. The two *RPGRIP1*^−L/−L^ Beagles, for which photopic and scotopic ERGs were performed, showed no cone response with reduced rod response. There were MLHDs with reduced rod ERG but no other clinical signs, and some dogs showed little or no fundus abnormality for at least 4.2 years after the detection of moderately to severely reduced ERG. By comparison, several dogs in the extended family studied herein did show dramatic changes in both fundus appearance and responses to functional tests over the same period. Thus it is likely that dogs with no apparent visual dysfunction on routine clinical assessment could develop visual deficits in later life. We hypothesize that *cord1* retinal degeneration starts early in life but the rate of progression varies, with some dogs not manifesting any dramatic change of the fundus appearance or overall visual function until much later.

In all five breeds in which the insertion occurs, a polymorphism in the downstream intron (marker: *RPGRIP1*_intron2) is the same for all chromosomes containing the insertion. In the Beagle, the shared haplotype region is limited to a maximum of 0.51 Mbp. The ERG observation of cone-rod photoreceptor degeneration in the two *RPGRIP1*^−L/−L^ Beagles supports the involvement of the *RPGRIP1* insertion in retinal phenotype. Hence, it is likely that the insertion appeared sometime in the past and was transmitted to several breeds as they were formed, with the adenine homopolymeric sequence being expanded in the Beagles, and demographic events leading to the increase in frequency of the insertion in MLHDs, ESSs, and Beagles.

Absence (or near absence) of ERG response in dogs with no apparent visual deficits was seen in one of the MLHDs with undetectable rod response (MLD4 of Family K) and in the Beagle with undetectable cone and severely reduced rod response. A similar observation has been made in some of the *RPGRIP1*^−/−^ MLHD from a second research colony founded from the one used by Mellersh et al. [18], and has also been described in the retinal degeneration associated with human nephronophthisis [40]. Given our observations, it is likely that some of the control dogs, determined on the basis of lack of apparent visual deficit, had retinal degeneration that could only be detected with ERG.

Although some genotype-phenotype discrepancies could be reduced by reconsidering the phenotype, and by functionally demonstrating the involvement of the *RPGRIP1* insertion in retinal degeneration in MLHDs, substantial discordances remain: the extensive range of onset among *RPGRIP1*^−/−^ PRA cases; and the 20.3% of PRA cases that are non-*RPGRIP1*^−/−^.

It is likely that PRA in the MLHD is oligogenic or additional loci are involved as modifiers giving rise to a variable age of onset, as has been suggested for *prcd* [7] and *XLPRA1* [41]. Such loci would have gone undetected in the original study if all the dogs in the colony were fixed for them due to a founder effect; this would explain the uniformity of the PRA phenotype in the colony MLHDs.

The 12 PRA-affected MLHDs which were non-*RPGRIP1*^−/−^ had dissimilar marker alleles in the *cord1* critical region compared to *RPGRIP1*^−/−^ PRA cases. Therefore, if each of these 12 cases is indeed affected with a genetic form of retinal degeneration, it is possible that other forms of PRA caused by different loci are present in this breed, albeit with lower prevalence than *cord1*. This could be the same for PRA affected non-*RPGRIP1*^−/−^ ESSs.

The mutation in *NPHP4* causing an early-onset cone-rod dystrophy in the SWHD [29] was not present in the MLHDs used in this study (data not shown). *NPHP4* could be a candidate modifier since mutations in either *RPGRIP1* or *NPHP4*, causing disruption of the interaction between the two gene products have been observed in LCA patients [42]. A microsatellite marker within *NPHP4* was not associated with PRA in MLHDs (data not shown).

Mutations known to cause PRA in other dog breeds could also be present in MLHDs as a result of interbreeding before or during breed formation. Known PRA-causing mutations in *PDE6B* [43], *RDS/Peripherin* and *ROM1* [44], and *PDE6A* [45] have previously been excluded in PRA-affected MLHDs, and we have also excluded the *prcd* mutation from the PRA cases of MLHDs used in this study (data not shown).

The RPGRIP1 protein was first identified through the interaction with the retinitis pigmentosa GTPase regulator (RPGR) protein [20] causing X-linked RP in humans and X-linked PRA in Samoyeds and Siberian Huskies [22]. Since the phenotypic variation in PRA cases of the MLHD had no association with gender (data not shown), it is unlikely that *RPGR* plays a role in the disease.

Given that a substantial proportion of *RPGRIP1*^−/−^ dogs appeared to retain functional vision, it could be argued that the RPGRIP1protein may not be essential in these dogs visual function. A homologous protein such as RPGRIP1L or other pathways could be compensating the luck of functional protein. However, the complete loss of *RPGRIP1* gene expression in *RPGRIP1*^−/−^ dogs remains putative, and further functional work is underway.

As animal models for gene therapy, it is important that the phenotype in the affected cases is predictable and consistent. However, as we have observed in this study, the phenotype of the PRA cases in pet MLHDs is not uniform: onset age and disease progression varies. Moreover, the genotype-phenotype discordance makes it difficult to predict the dog’s phenotype solely by the presence of the *RPGRIP1* insertion. Although the effort to understand the entire picture of the disease is underway, we do not have the molecular means to allow an accurate prediction of the phenotype to date. The factor that affects the variable disease expression must be elucidated before cases from the pet MLHD population can be considered ideal animal models. The relative phenotypic uniformity in the research dogs could be attributed to a homogeneous genetic background due to inbreeding, and common environmental factors.

In conclusion, the phenotypic variation and the discordance with the insertion in exon 2 of *RPGRIP1* observed here indicate that the cause of PRA in the MLHD pet population is more complex than initially thought (i.e., a single gene, fully-penetrant Mendelian trait segregating in a single breed). Although the complete association of the *RPGRIP1* insertion with PRA in MLHDs remains to be established, observations of cone-rod retinal degeneration in *RPGRIP1*^−L/−L^ Beagles indicate its involvement in retinal degeneration. Yet additional as well as alternative loci would be required to account for the extensive phenotypic variation in addition to the remaining genotype-phenotype discordance. Further studies are under way to obtain the full genetic picture of PRA in MLHDs.

## References

[1] Petersen-Jones S Advances in the molecular understanding of canine retinal diseases. J Small Anim Pract 2005 46 371 80 16119056 10.1111/j.1748-5827.2005.tb00333.x

[2] Neitz J Geist T Jacobs GH Color vision in the dog. Vis Neurosci 1989 3 119 25 2487095 10.1017/s0952523800004430

[3] Mowat FM Petersen-Jones SM Williamson H Williams DL Luthert PJ Ali RR Bainbridge JW Topographical characterization of cone photoreceptors and the area centralis of the canine retina. Mol Vis 2008 14 2518 27 19112529 PMC2610288

[4] McGreevy P Grassi TD Harman AM A strong correlation exists between the distribution of retinal ganglion cells and nose length in the dog. Brain Behav Evol 2004 63 13 22 14673195 10.1159/000073756

[5] Dekomien G Runte M Godde R Epplen JT Generalized progressive retinal atrophy of Sloughi dogs is due to an 8-bp insertion in exon 21 of the PDE6B gene. Cytogenet Cell Genet 2000 90 261 7 11124530 10.1159/000056785

[6] Suber ML Pittler SJ Qin N Wright GC Holcombe V Lee RH Craft CM Lolley RN Baehr W Hurwitz RL Irish setter dogs affected with rod/cone dysplasia contain a nonsense mutation in the rod cGMP phosphodiesterase beta-subunit gene. Proc Natl Acad Sci USA 1993 90 3968 72 8387203 10.1073/pnas.90.9.3968 PMC46427

[7] Zangerl B Goldstein O Philp AR Lindauer SJ Pearce-Kelling SE Mullins RF Graphodatsky AS Ripoll D Felix JS Stone EM Acland GM Aguirre GD Identical mutation in a novel retinal gene causes progressive rod-cone degeneration in dogs and retinitis pigmentosa in humans. Genomics 2006 88 551 63 16938425 10.1016/j.ygeno.2006.07.007 PMC3989879

[8] Kennan A Aherne A Humphries P Light in retinitis pigmentosa. Trends Genet 2005 21 103 10 15661356 10.1016/j.tig.2004.12.001

[9] Hartong DT Berson EL Dryja TP Retinitis pigmentosa. Lancet 2006 368 1795 809 17113430 10.1016/S0140-6736(06)69740-7

[10] Stone EM Leber congenital amaurosis - a model for efficient genetic testing of heterogeneous disorders: LXIV Edward Jackson Memorial Lecture. Am J Ophthalmol 2007 144 791 811 17964524 10.1016/j.ajo.2007.08.022

[11] den Hollander AI Roepman R Koenekoop RK Cremers FP Leber congenital amaurosis: genes, proteins and disease mechanisms. Prog Retin Eye Res 2008 27 391 419 18632300 10.1016/j.preteyeres.2008.05.003

[12] Boon CJ den Hollander AI Hoyng CB Cremers FP Klevering BJ Keunen JE The spectrum of retinal dystrophies caused by mutations in the peripherin/RDS gene. Prog Retin Eye Res 2008 27 213 35 18328765 10.1016/j.preteyeres.2008.01.002

[13] Zernant J Külm M Dharmaraj S den Hollander AI Perrault I Preising MN Lorenz B Kaplan J Cremers FP Maumenee I Koenekoop RK Allikmets R Genotyping microarray (disease chip) for Leber congenital amaurosis: detection of modifier alleles. Invest Ophthalmol Vis Sci 2005 46 3052 9 16123401 10.1167/iovs.05-0111

[14] Yzer S Leroy BP De Baere E de Ravel TJ Zonneveld MN Voesenek K Kellner U Ciriano JP de Faber JT Rohrschneider K Roepman R den Hollander AI Cruysberg JR Meire F Casteels I van Moll-Ramirez NG Allikmets R van den Born LI Cremers FP Microarray-based mutation detection and phenotypic characterization of patients with Leber congenital amaurosis. Invest Ophthalmol Vis Sci 2006 47 1167 76 16505055 10.1167/iovs.05-0848

[15] Yzer S Fishman GA Racine J Al-Zuhaibi S Chakor H Dorfman A Szlyk J Lachapelle P van den Born LI Allikmets R Lopez I Cremers FP Koenekoop RK CRB1 heterozygotes with regional retinal dysfunction: implications for genetic testing of leber congenital amaurosis. Invest Ophthalmol Vis Sci 2006 47 3736 44 16936081 10.1167/iovs.05-1637

[16] Curtis R Barnett KC Progressive retinal atrophy in miniature longhaired dachshund dogs. Br Vet J 1993 149 71 85 8439801 10.1016/S0007-1935(05)80211-8

[17] Turney C Chong NH Alexander RA Hogg CR Fleming L Flack D Barnett KC Bird AC Holder GE Luthert PJ Pathological and electrophysiological features of a canine cone-rod dystrophy in the miniature longhaired dachshund. Invest Ophthalmol Vis Sci 2007 48 4240 9 17724213 10.1167/iovs.04-0737

[18] Lhériteau E Libeau L Stieger K Deschamps JY Mendes-Madeira A Provost N Lemoine F Mellersh C Ellinwood NM Cherel Y Moullier P Rolling F The RPGRIP1-deficient dog, a promising canine model for gene therapy. Mol Vis 2009 15 349 61 19223988 PMC2642837

[19] Mellersh CS Boursnell ME Pettitt L Ryder EJ Holmes NG Grafham D Forman OP Sampson J Barnett KC Blanton S Binns MM Vaudin M Canine RPGRIP1 mutation establishes cone-rod dystrophy in miniature longhaired dachshunds as a homologue of human Leber congenital amaurosis. Genomics 2006 88 293 301 16806805 10.1016/j.ygeno.2006.05.004

[20] Boylan JP Wright AF Identification of a novel protein interacting with RPGR. Hum Mol Genet 2000 9 2085 93 10958647 10.1093/hmg/9.14.2085

[21] Roepman R Bernoud-Hubac N Schick DE Maugeri A Berger W Ropers HH Cremers FP Ferreira PA The retinitis pigmentosa GTPase regulator (RPGR) interacts with novel transport-like proteins in the outer segments of rod photoreceptors. Hum Mol Genet 2000 9 2095 105 10958648 10.1093/hmg/9.14.2095

[22] Zhang Q Acland GM Wu WX Johnson JL Pearce-Kelling S Tulloch B Vervoort R Wright AF Aguirre GD Different RPGR exon ORF15 mutations in Canids provide insights into photoreceptor cell degeneration. Hum Mol Genet 2002 11 993 1003 11978759 10.1093/hmg/11.9.993

[23] Pawlyk BS Smith AJ Buch PK Adamian M Hong DH Sandberg MA Ali RR Li T Gene replacement therapy rescues photoreceptor degeneration in a murine model of Leber congenital amaurosis lacking RPGRIP. Invest Ophthalmol Vis Sci 2005 46 3039 45 16123399 10.1167/iovs.05-0371

[24] Zhao Y Hong DH Pawlyk B Yue G Adamian M Grynberg M Godzik A Li T The retinitis pigmentosa GTPase regulator (RPGR)- interacting protein: subserving RPGR function and participating in disk morphogenesis. Proc Natl Acad Sci USA 2003 100 3965 70 12651948 10.1073/pnas.0637349100 PMC153031

[25] Hong DH Yue G Adamian M Li T Retinitis pigmentosa GTPase regulator (RPGRr)-interacting protein is stably associated with the photoreceptor ciliary axoneme and anchors RPGR to the connecting cilium. J Biol Chem 2001 276 12091 9 11104772 10.1074/jbc.M009351200

[26] Dryja TP Adams SM Grimsby JL McGee TL Hong DH Li T Andréasson S Berson EL Null RPGRIP1 alleles in patients with Leber congenital amaurosis. Am J Hum Genet 2001 68 1295 8 11283794 10.1086/320113 PMC1226111

[27] Hameed A Abid A Aziz A Ismail M Mehdi SQ Khaliq S Evidence of RPGRIP1 gene mutations associated with recessive cone-rod dystrophy. J Med Genet 2003 40 616 9 12920076 10.1136/jmg.40.8.616 PMC1735563

[28] Booij JC Florijn RJ ten Brink JB Loves W Meire F van Schooneveld MJ de Jong PT Bergen AA Identification of mutations in the AIPL1, CRB1, GUCY2D, RPE65, and RPGRIP1 genes in patients with juvenile retinitis pigmentosa. J Med Genet 2005 42 e67 16272259 10.1136/jmg.2005.035121 PMC1735944

[29] Wiik AC Wade C Biagi T Ropstad EO Bjerkås E Lindblad-Toh K Lingaas F A deletion in nephronophthisis 4 (NPHP4) is associated with recessive cone-rod dystrophy in standard wire-haired dachshund. Genome Res 2008 18 1415 21 18687878 10.1101/gr.074302.107 PMC2527698

[30] Acland GM Aguirre GD Ray J Zhang Q Aleman TS Cideciyan AV Pearce-Kelling SE Anand V Zeng Y Maguire AM Jacobson SG Hauswirth WW Bennett J Gene therapy restores vision in a canine model of childhood blindness. Nat Genet 2001 28 92 5 11326284 10.1038/ng0501-92

[31] Cai X Conley SM Naash MI RPE65: role in the visual cycle, human retinal disease, and gene therapy. Ophthalmic Genet 2009 30 57 62 19373675 10.1080/13816810802626399 PMC2821785

[32] Tao W Application of encapsulated cell technology for retinal degenerative diseases. Expert Opin Biol Ther 2006 6 717 26 16805711 10.1517/14712598.6.7.717

[33] Barnett KC Retinal atrophy. Vet Rec 1965 77 1543 60 5321572

[34] Diau GY Loew ER Wijendran V Sarkadi-Nagy E Nathanielsz PW Brenna JT Docosahexaenoic and arachidonic acid influence on preterm baboon retinal composition and function. Invest Ophthalmol Vis Sci 2003 44 4559 66 14507905 10.1167/iovs.03-0478

[35] Narfström K Ekesten B Rosolen SG Spiess BM Percicot CL Ofri R Committee for a Harmonized ERG Protocol, European College of Veterinary Ophthalmology. Guidelines for clinical electroretinography in the dog. Doc Ophthalmol 2002 105 83 92 12462438 10.1023/a:1020524305726

[36] Benson G Tandem repeats finder: a program to analyze DNA sequences. Nucleic Acids Res 1999 27 573 80 9862982 10.1093/nar/27.2.573 PMC148217

[37] Rozen S Skaletsky H Primer3 on the WWW for general users and for biologist programmers. Methods Mol Biol 2000 132 365 86 10547847 10.1385/1-59259-192-2:365

[38] Sham PC Curtis D Monte Carlo tests for associations between disease and alleles at highly polymorphic loci. Ann Hum Genet 1995 59 97 105 7762987 10.1111/j.1469-1809.1995.tb01608.x

[39] Sidák Z Rectangular confidence regions for the means of multivariate normal distributions. J Am Stat Assoc 1967 62 626 33

[40] Dufier JL Orssaud D Dhermy P Gubler MC Gagnadoux MF Kleinknecht C Broyer M Ocular changes in some progressive hereditary nephropathies. Pediatr Nephrol 1987 1 525 30 3153327 10.1007/BF00849264

[41] Guyon R Pearce-Kelling SE Zeiss CJ Acland GM Aguirre GD Analysis of six candidate genes as potential modifiers of disease expression in canine XLPRA1, a model for human X-linked retinitis pigmentosa 3. Mol Vis 2007 13 1094 105 17653054 PMC2779147

[42] Roepman R Letteboer SJ Arts HH van Beersum SE Lu X Krieger E Ferreira PA Cremers FP Interaction of nephrocystin-4 and RPGRIP1 is disrupted by nephronophthisis or Leber congenital amaurosis-associated mutations. Proc Natl Acad Sci USA 2005 102 18520 5 16339905 10.1073/pnas.0505774102 PMC1317916

[43] Clements PJ Gregory CY Peterson-Jones SM Sargan DR Bhattacharya SS Confirmation of the rod cGMP phosphodiesterase beta subunit (PDE beta) nonsense mutation in affected rcd-1 Irish setters in the UK and development of a diagnostic test. Curr Eye Res 1993 12 861 6 8261797 10.3109/02713689309020391

[44] Runte M Dekomien G Epplen JT Evaluation of RDS/Peripherin and ROM1 as candidate genes in generalised progressive retinal atrophy and exclusion of digenic inheritance. Anim Genet 2000 31 223 7 10895316 10.1046/j.1365-2052.2000.00633.x

[45] Dekomien G Epplen JT Exclusion of the PDE6A gene for generalised progressive retinal atrophy in 11 breeds of dog. Anim Genet 2000 31 135 9 10782214 10.1046/j.1365-2052.2000.00611.x

[46] Guyon R Lorentzen TD Hitte C Kim L Cadieu E Parker HG Quignon P Lowe JK Renier C Gelfenbeyn B Vignaux F DeFrance HB Gloux S Mahairas GG André C Galibert F Ostrander EA A 1-Mb resolution radiation hybrid map of the canine genome. Proc Natl Acad Sci USA 2003 100 5296 301 12700351 10.1073/pnas.0831002100 PMC154339

